# COVID-19-Associated Pulmonary Aspergillosis in a Patient Treated With Remdesivir, Dexamethasone, and Baricitinib: A Case Report

**DOI:** 10.7759/cureus.23755

**Published:** 2022-04-02

**Authors:** Ayako Shimada, Shinnosuke Ohnaka, Kosumi Kubo, Masanao Nakashima, Atsushi Nagai

**Affiliations:** 1 Department of Respiratory Medicine, Shin-Yurigaoka General Hospital, Kanagawa, JPN

**Keywords:** jak inhibitor, baricitinib, pulmonary aspergillosis, respiratory failure, covid-19

## Abstract

Remdesivir, dexamethasone, and baricitinib have recently been used to treat patients with coronavirus disease 2019 (COVID-19) and respiratory failure. However, the adverse effects of combination therapy have not been fully explored. A 64-year-old man was diagnosed with COVID-19 and was treated with remdesivir, dexamethasone, and baricitinib. His respiratory condition worsened on day 17, and in the following days, he was diagnosed with pneumomediastinum and COVID-19-associated pulmonary aspergillosis (CAPA). His condition improved with a reduction in the corticosteroid regime and antifungal treatment. This is the first case of pulmonary aspergillosis in a patient with COVID-19 that was treated with remdesivir, dexamethasone, and baricitinib.

## Introduction

Secondary infections (superinfections) are known complications of the coronavirus disease 2019 (COVID-19) [1]. COVID-19-associated pulmonary aspergillosis (CAPA) is an infectious complication that has been increasingly reported worldwide. A systematic review of CAPA in critical or intensive care unit (ICU)-treated COVID-19 patients has reported the incidence and mortality to be 10.2% and 54.9%, respectively. However, the incidence varies widely among reports [2], and the high mortality rate highlights the importance of early diagnosis and adequate treatment of Aspergillus infection. Several factors, including viral-associated airway epithelial cell injury, immunosuppression caused by corticosteroids or other immunomodulatory drug use, host immunodeficiency, and temporary facilities with inadequate room ventilation, are considered risk factors for CAPA [3-5].

However, it is important to control the hyperinflammatory state in patients with COVID-19 and acute respiratory failure or multiple organ dysfunction. Drugs such as dexamethasone and baricitinib, selective Janus kinase (JAK) inhibitors, are effective in COVID-19 patients who require oxygen therapy or mechanical ventilation [6-8]. Although randomized trials of baricitinib with the standard of care, including corticosteroids, showed no differences in infectious complications [8], there is still little information on the complications of immunomodulatory combinations in COVID-19 patients.

Herein, we present the first report of a case of a patient who developed pulmonary aspergillosis during remdesivir, dexamethasone, and baricitinib therapy for COVID-19.

## Case presentation

**Figure 1:**
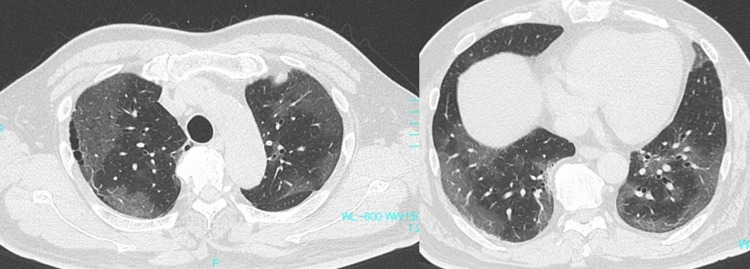
Chest CT findings on admission Bilateral ground glass opacity in peripheral lungs were seen upon admission.

A 64-year-old man with fever and dyspnea was admitted to our hospital in November 2021. The patient was symptomatic three days before admission and was diagnosed with COVID-19 based on the antigen test for severe acute respiratory syndrome coronavirus 2 (SARS-CoV-2) the day before. The patient had untreated hypertension and diabetes mellitus (DM). He had a 40 pack-year history of smoking; however, he had quit smoking 20 years ago. On admission, his body mass index was 29.7 (bodyweight, 92 kg), body temperature was 36.9 °C, respiratory rate was 24 breaths/min, and oxygen saturation in room air was 88%. His breath sounds were normal on auscultation, and physical examination results were unremarkable. Chest computed tomography (CT) revealed diffuse bilateral ground-glass opacities (chest CT severity score: 20 [9]) (Figure 1).

**Figure 2:**
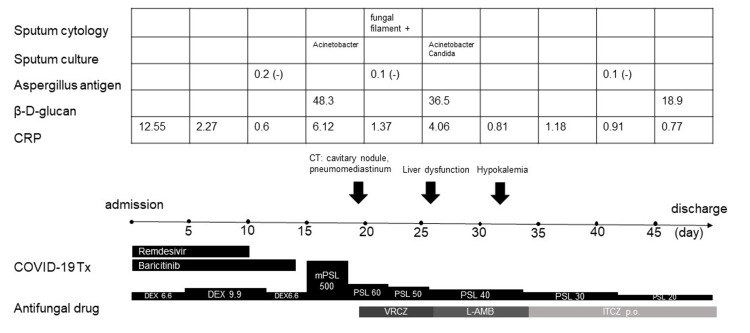
Clinical course of the presented case CRP: C-reactive protein; CT: computed tomography; DEX: dexamethasone; ITCZ: itraconazole; L-AMB: liposomal amphotericin B; mPSL: methylprednisolone; PSL: prednisolone; Tx: treatment; VRCZ: voriconazole

Laboratory tests revealed elevated serum lactate dehydrogenase levels (451 U/L), elevated C-reactive protein levels (12.55 mg/dL), elevated white blood cell counts (11,400/µL), neutrophil counts (10,249/µL), elevated liver enzymes (aspartate aminotransferase 96 U/L, alanine aminotransferase 67 U/L), elevated hemoglobin A1c levels (7.6%), and decreased sodium carbonate levels (130 mEq/L). His renal function and D-dimer levels were normal. COVID-19 treatment with remdesivir, dexamethasone, and baricitinib was initiated (Figure 2).

The patient required high-flow nasal oxygen therapy (HFNO) with maximum oxygenation of 75% on day five, and his respiratory condition gradually resolved.

**Figure 3:**
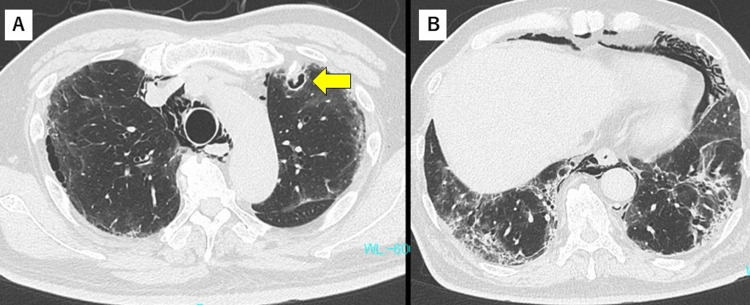
Chest CT findings on day 19 (A) Cavity seen with a small nodule in the left upper lobe (arrow). (B) Pneumomediastinum and fibrosis in lower lungs.

However, on day 17, dyspnea and oxygenation worsened. Chest radiography results revealed augmentation of bilateral infiltration in the lower lungs. Secondary bacterial pneumonia or worsening of pneumonia due to SARS-CoV-2 was suspected, and antibiotic treatment with ceftriaxone and high-dose corticosteroid administration (intravenous methylprednisolone at a dose of 500 mg for three days, followed by prednisolone at a dose of 0.85 mg/kg) was initiated. Chest CT images acquired on day 19 showed pneumomediastinum and cavitary lesions in the left upper lung (Figure 3A) and fibrosis in the lower lungs (Figure 3B).

**Figure 4:**
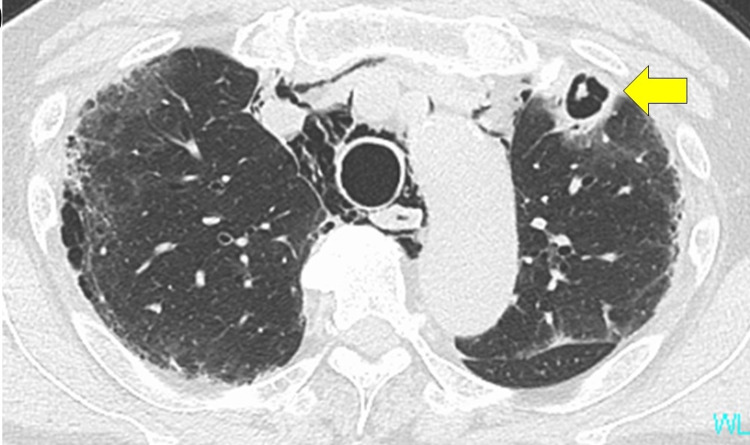
Chest CT findings on day 26 Cavity in the upper lobe enlarged (arrow).

CAPA was suspected, and intravenous administration of voriconazole (at a loading dose of 500mg twice daily on day one, followed by 300mg twice daily on days 2 to 7) was initiated. The serum β-D-glucan test result was positive, and sputum cytology revealed fungal filaments. Serum antigen and sputum cultures were negative for Aspergillus. With rest and corticosteroid tapering, pneumomediastinum improved. Voriconazole-induced acute liver dysfunction was observed on day 26, and the antifungal drug was switched to amphotericin B (300mg once daily, intravenously). On day 34, amphotericin B was switched to itraconazole (200mg twice daily, orally) because of hypokalemia (minimum 2.3mEq/L). The cavity enlarged on the follow-up CT, which was done on day 26 (Figure 4). 

**Figure 5:**
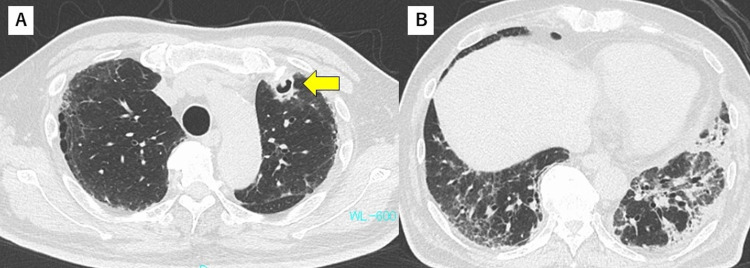
Chest CT findings on day 42 (A) Gradual improvement of the cavity was observed (arrow). (B) Lung fibrosis improved with partial lung shrinkage.

However, CT scan images before discharge showed a reduction in the cavitary lung lesion (Figure 5).

The patient’s respiratory condition and electrolyte abnormalities gradually improved, and he was discharged from the hospital on day 46.

## Discussion

To the best of our knowledge, this is the first report of CAPA in a patient treated with a combination of remdesivir, dexamethasone, and baricitinib.

Pulmonary aspergillosis is often difficult to diagnose in critically ill patients because radiological changes are nonspecific, and serum galactomannan (GM) has low sensitivity. Moreover, the gold standard for obtaining mycological evidence of invasive diseases is bronchoscopy, which is often avoided to protect healthcare workers from aerosol exposure. The consensus definition for CAPA has been proposed by the European Respiratory Society (ERS) and the European Confederation for Medical Mycology, International Society for Human and Animal Mycology [10,11]. To avoid an overestimation of CAPA due to Aspergillus contamination or colonization, both criteria suggest bronchoalveolar lavage (BAL) or non-bronchoscopic lavage acquire positive results for Aspergillus culture or GM. However, due to the high mortality rate of CAPA, these two criteria propose active screening for CAPA. In the ERS criteria, when BAL is not possible, the presence of at least one serum biomarker, such as Aspergillus antigen, polymerase chain reaction, or β-D-glucan positivity, indicates CAPA.

In our report, the patient’s chest CT images showed a cavitary lesion, which is a classical finding of invasive aspergillosis. His sputum culture was negative for Aspergillus; however, it showed microscopic fungal elements, and his serum β-D-glucan test was positive. The BAL specimen was not collected because the patient had a severe respiratory failure due to pneumomediastinum at the onset of putative CAPA. The cavitary lesion on chest CT and β-D-glucan test values both showed improvement after antifungal treatment, which supports the diagnosis of CAPA. Our patient had diabetes mellitus and severe respiratory distress and was treated with high-dose corticosteroids, all of which can be risk factors for Aspergillus infection. It was quite difficult to assess the involvement of baricitinib in the present case.

Baricitinib inhibits the JAK-STAT pathway, downregulates multiple inflammatory cytokines, and inhibits viral entry via the AAK1 receptor [12]. The COV-BARRIER study showed that treatment with baricitinib, in addition to the standard of care (including dexamethasone), reduced mortality [8]. In Japan, this regimen was approved for the treatment of COVID-19 patients with respiratory failure in April 2021. Since then, triple therapy with remdesivir, dexamethasone, and baricitinib has been widely used in Japan [13]. Although the COV-BARRIER study reported that baricitinib-added therapy did not increase serious infections, there are still concerns that JAK inhibitors increase the incidence of CAPA when used with corticosteroids. In vitro and in vivo experiments have shown reduced fungal elimination by the JAK inhibitor ruxoltinib via inhibition of Interleukin-6/23-activated neutrophils [14]. However, it is unclear how the combination of baricitinib and corticosteroids affects immunity against fungi.

## Conclusions

We treated a COVID-19 patient who developed pulmonary Aspergillus infection after treatment with remdesivir, dexamethasone, and baricitinib. Combination therapy with immunomodulatory drugs has been commonly used in patients with severe COVID-19, but the incidence and characteristics of CAPA in these patients are unclear. Physicians should be aware of CAPA, and early screening should be performed for worsening respiratory conditions in patients with COVID-19.
